# Supplementary material for: Zebrafish imaging reveals TP53 mutation switching oncogene-induced senescence from suppressor to driver in primary tumorigenesis
Source: Nat Commun. 2022 Mar 18;13:1417. doi: 10.1038/s41467-022-29061-6 (PMC8933407; doi:10.1038/s41467-022-29061-6)
Supplement: Supplementary file 1 — Supplementary Information [file 41467_2022_29061_MOESM1_ESM.pdf]

Zebrafish imaging reveals TP53 mutation switching oncogene-induced senescence from suppressor to driver in primary tumorigenesis

Haraoka et al.

Supplementary information:
Supplementary Figs. 1–13 and legends
Supplementary Table 1

Supplementary Fig. 1

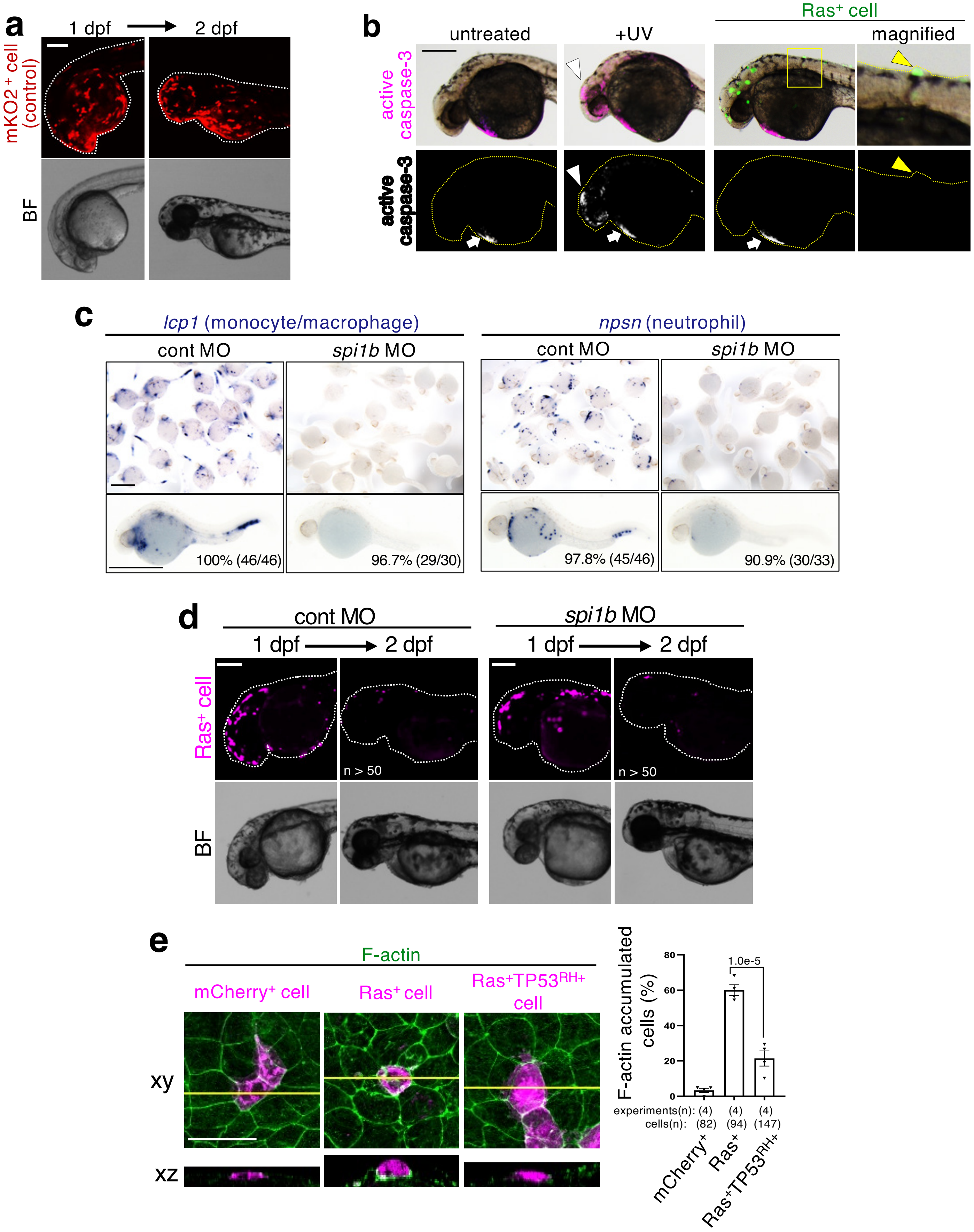

Supplementary Fig. 1. Immune cell-independent elimination of oncogenic cells.

a Low-dose injection of UAS-driven plasmids induces mosaic distribution of fluorescent cells, each of which are surrounded by normal cells in the larval skin. Representative fluorescent and bright field (BF) images show the larvae with $mKO2^{+}$ cells (red). Dot lines surround larvae. Scale bar: 200 µm. **b** Apoptosis is not involved in eliminating Ras cells. Representative images show active caspase-3 (upper panels; magenta, lower panels; grey) in 1 dpf larvae untreated, treated with ultraviolet (UV), or with cells expressing Ras^{G12V} and GFP (Ras^{+} cell; green). As a positive control for active caspase-3 detection, larvae were exposed to UV (60 mJ/cm^{2}) and incubated for 5 h at 28.5°C. Dot lines surround larvae. Magnification of boxed area (yellow line). White arrowhead indicates apoptotic cells, and white arrows indicate apoptotic cells generated in physiological condition. Yellow arrowheads indicate protruded Ras^{+} cells. Scale bar: 200 µm. **c** *spi1b* MO depletes innate immune cells. *in situ* hybridization of *lcp1* (monocyte and macrophage marker gene, left) and *npsn* (neutrophil marker gene, right) in larvae with control MO or *spi1b* MO. Larvae percentages and numbers with similar expression patterns are shown. Scale bar: 500 µm. $p <$ 0.0001 for control MO versus *spi1b* MO in both *lcp1* and *npsn* (Fisher's exact test with Benjamini–Hochberg correction). **d** Immune cells are not required to obliterate mosaic Ras^{G12V} cells. Representative images show the larvae with $mKO2^{+}Ras^{G12V}$ (Ras^{+}) cells (magenta) with control MO (n > 50) or *spi1b* MO (n > 50). Dot lines surround larvae. Scale bar: 200 µm. Note that almost all larvae with *spi1b* MO showed a phenotype similar to those with control MO. **e** Representative confocal images show F-actin (green) in mosaic cells expressing mCherry alone (control) or with Ras^{G12V}, or both Ras^{G12V} and $TP53^{R175H}$ ($mCherry^{+}$, Ras^{+}, $Ras^{+}TP53^{RH+}$ cells) (magenta). Scale bar: 20 µm. Bar graph shows the percentage of GFP^{+} cells with F-actin accumulated cells (mean ± SEM). Each dot represents one larva. A two-tailed one-way ANOVA test with Sidak correction was used. Source data are provided as a Source Data file.

Supplementary Fig. 2

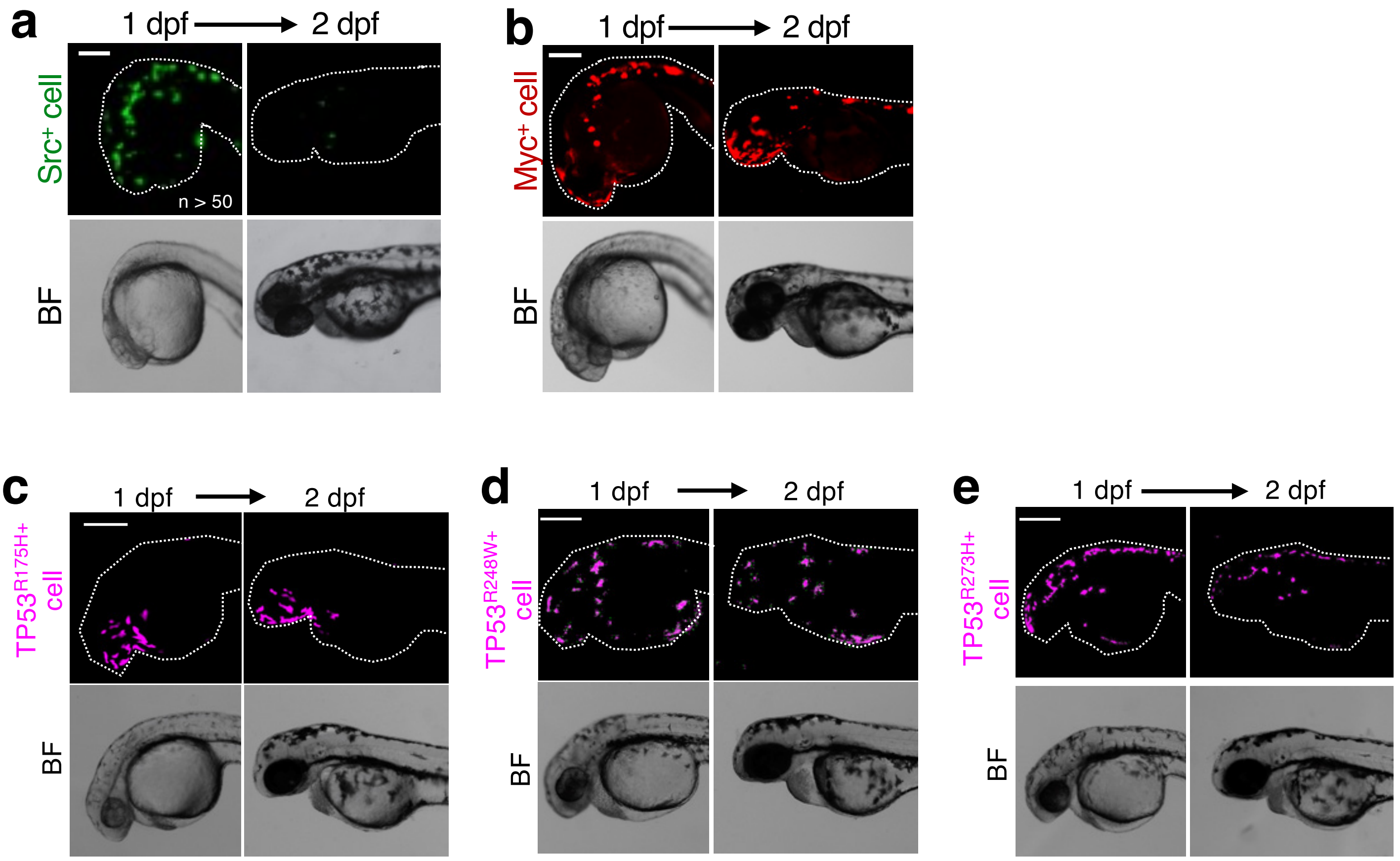

Supplementary Fig. 2. A specific type of oncogenic activation triggers cell elimination. **a** Mosaic v-Src cell disappears from larval skin. Representative images show the larvae with mosaically introduced GFP^+v-Src (Src^+) cells (green). Dot lines surround larvae. Scale bar: 200 μm. **b** Mosaically Myc-high cells remain in larval skin. Representative fluorescent and BF images show the larvae with mosaically introduced $mKO2^+$ cells overexpressing Myc (Myc^+) (red). Dot lines surround larvae. Scale bar: 200 μm. **c**–**e** Mosaic TP53 mutant cells remain in larval skin. Representative fluorescent and BF images show the larvae with mosaically introduced $mKO2^+$ cells overexpressing gain-of function TP53 mutants (**c**; $TP53^{R175H+}$, **d**; $TP53^{R248W+}$, **e**; $TP53^{R273H+}$) (magenta). Dot lines surround larvae. Scale bar: 200 μm.

Supplementary Fig. 3

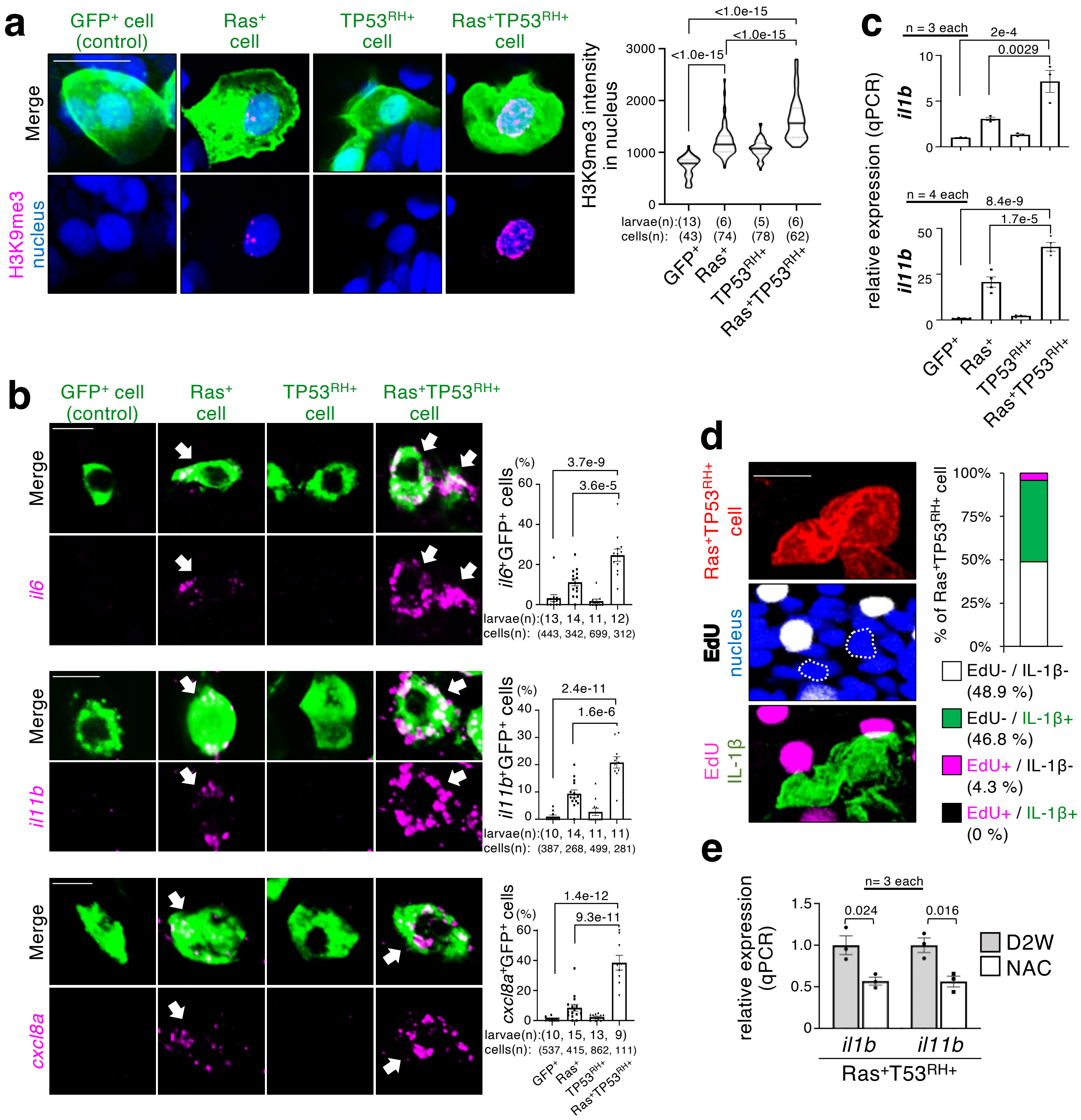

Supplementary Fig. 3. Ras^{G12V} and TP53^{R175H} mutations synergistically stimulate cellular senescence.

a Mosaic Ras^{G12V} or Ras^{G12V}TP53^{R175H} cells express senescence markers, trimethyl-histone H3 (Lys9), H3K9me3 (magenta) in their nuclei (blue). Representative confocal images show mosaically introduced GFP^{+}, Ras^{+}, TP53^{RH+}, or Ras^{+}TP53^{RH+} cells (green). Scale bar: 20 µm. Violin plots of H3K9me3 intensities in their nucleus of GFP^{+} cells show 75th, 50th (median), and 25th percentiles. **b, c** Ras^{G12V} and TP53^{R175H} synergistically promote the expression of SASP factors. In **b**, Representative confocal images show fluorescent *in situ* hybridization of SASP factors (interleukin-6 (*il6)* mRNA*,* interleukin-11b (*il11b*) mRNA*,* and interleukin-8 (*cxcl8a*) mRNA) (magenta) in mosaically introduced GFP^{+}, Ras^{+}, TP53^{RH+}, or Ras^{+}TP53^{RH+} cells (green) in the larvae. Scale bar: 10 µm. Right bar graph shows percentage of GFP^{+} cells with SASP factors positive cells (mean ± SEM). Each dot represents one larva. A two-tailed one-way ANOVA test with Sidak correction was used. In **c**, qPCR analysis for expression of interleukin-1β (*il1b*) and *il11b*. Bar graph shows percentage of relative expression of *il1b* or *il11b* (mean ± SEM). A two-tailed one-way ANOVA test with Sidak correction was used. Each dot represents an independent experimental result. **d** Cell cycle arrested Ras^{G12V+}TP53^{R175H+}cells express IL-1β. Representative confocal images (left panels) show EdU staining of mosaically introduced mKO2^{+}Ras^{G12V}TP53^{R175H} (Ras^{+}TP53^{RH+}) cells (red) in Tg (*il1b*:egfp) larvae. Middle panel (EdU-positive cells as grey and nucleus as blue; Dot lines surround the nucleus of Ras^{+}TP53^{RH+} cells) indicates that Ras^{+}TP53^{RH+} cells were cell-cycle arrested cells. The bottom panel indicates that these arrested Ras^{+}TP53^{RH+} cells had upregulated IL-1β expression. Scale bar: 20 µm. EdU^{+}/IL-1β^{+} cells were not detected. **e** Reactive oxygen species (ROS) facilitates the expression of *il1b* and *il11b* in Ras^{G12V}TP53^{R175H} (Ras^{+}TP53^{RH+} cells) cells. qPCR analysis shows NAC treatment inhibits expression of SASP factors, *il1b* and *il11b*, in larvae with mosaic Ras^{+}TP53^{RH+} cells. Bar graph shows percentage of relative expression of *il1b* or *il11b* (mean± SEM). Each dot represents an independent experimental result. Unpaired two-tailed t-test was used. Source data are provided as a Source Data file.

Supplementary Fig. 4

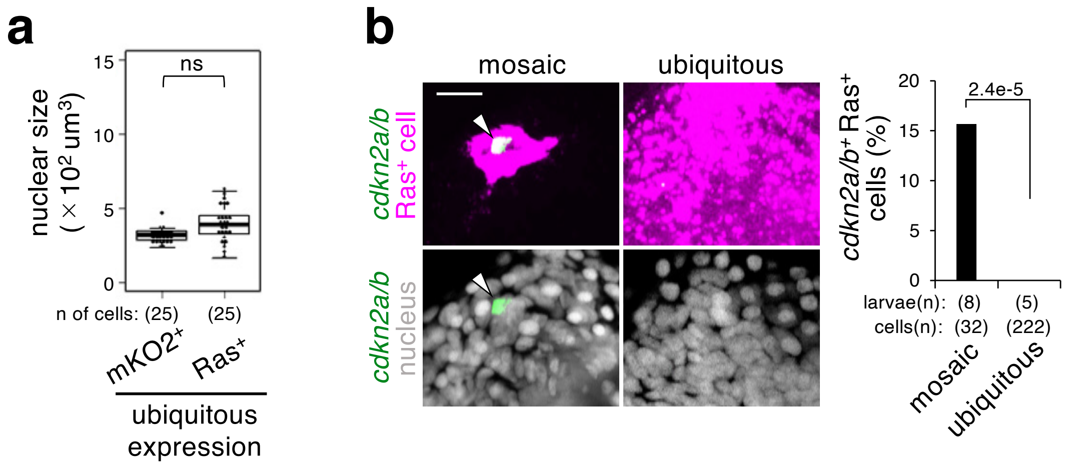

Supplementary Fig. 4. Ras^{G12V} cells senesce through communication with surrounding normal cells.

a Box plots of estimated nuclear size (μm^3) in ubiquitous expression of mKO2 alone and with Ras^{G12V} show first and third quartile, median is represented by a line, whiskers indicate the minimum and maximum, and outliers are shown as dots outside of the box. Each dot represents one cell. p = 0.18. Unpaired two-tailed t-test was used. **b** Representative confocal images show fluorescence *in situ* hybridization of *cdkn2a/b* mRNA (green) and *mKO2* mRNA (magenta), and nucleus (grey) in Tg(*krt4p:gal4*) larvae with mosaically introduced cells expressing Ras or Tg(*krt4p:gal4*; UAS:GAP43mKO2-T2A-H-Ras^{G12V}) larvae, which expressed Ras ubiquitously in the whole skin. Arrowhead indicates *cdkn2a/b*-expressing cells. Scale bar: 20 μm. Graphs show percentages of Ras$^+$ cells with *cdkn2a/b* expression. Fisher's exact test with Benjamini–Hochberg correction was used. Source data are provided as a Source Data file.

Supplementary Fig. 5

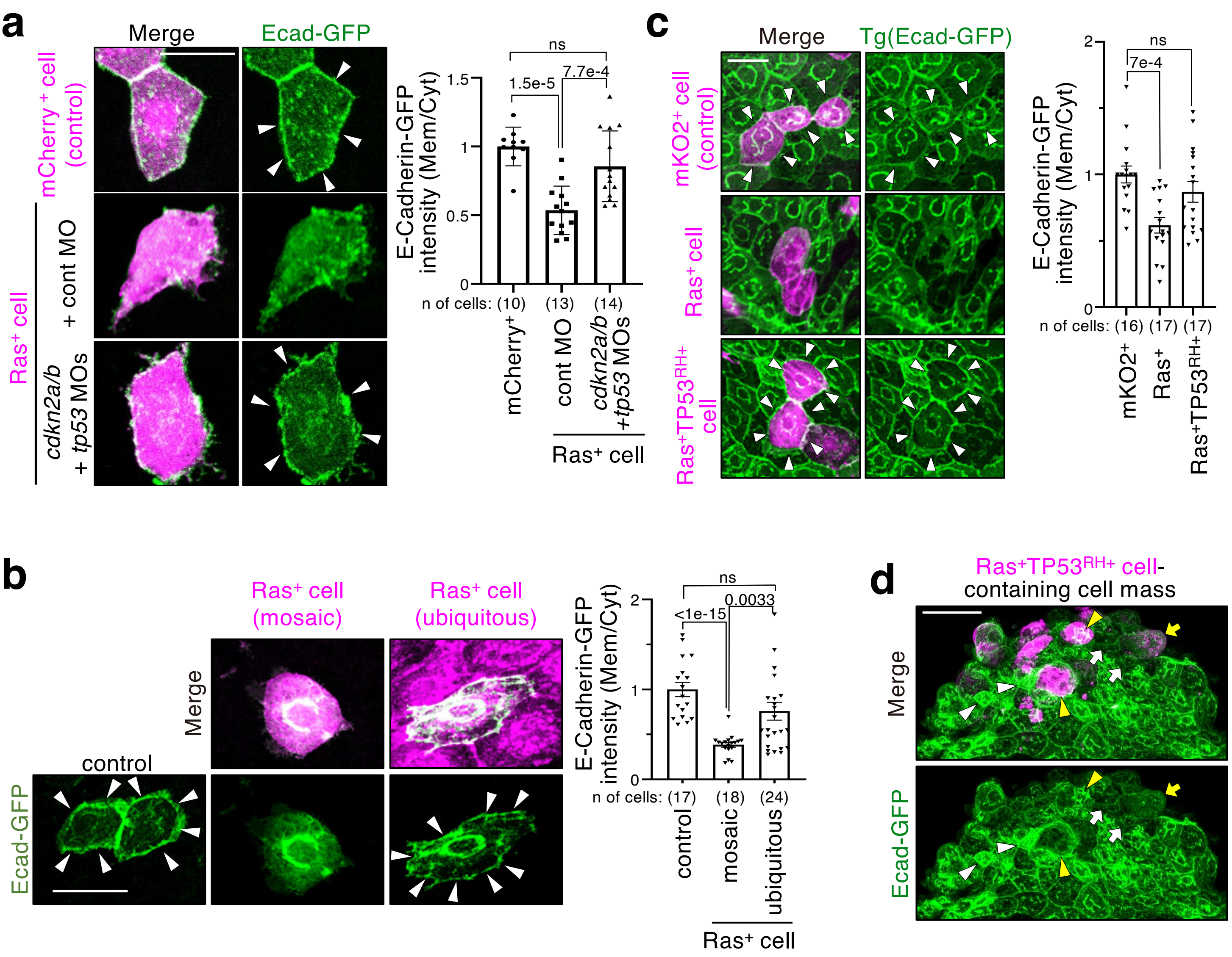

Supplementary Fig. 5. Mosaic introduction of Ras^{G12V} mutation reduces membrane E-cadherin levels, while additional TP53^{R175H} mutation prevents this reduction.

a Senesced Ras^{G12V} cells reduce the E-cadherin levels in their membrane. Representative confocal images show zebrafish E-cadherin-GFP (Ecad-GFP) expression (green) in larvae with mosaically introduced cells expressing mCherry alone (control) with or without Ras^{G12V} cells (mCherry^{+} or Ras^{+} cells) (magenta) and injected with control MO or *cdkn2a*/b and *tp53* MOs. Scale bar: 20 µm. Right graphs show the ratio of membrane/cytoplasmic E-cadherin-GFP intensity (mean ± SEM). Each dot represents one cell. A two-tailed one-way ANOVA test with Sidak correction was used. **b** Ras^{G12V+} cells reduce membrane E-cadherin levels by communicating with surrounding normal cells. Representative confocal images show Ecad-GFP (green) in the larvae with ubiquitously-introduced mKO2 alone cell (control) or mosaically- or ubiquitously-introduced mKO2^{+}Ras^{+} cells (magenta). Scale bar: 20 µm. Right graphs show the ratio of membrane/cytoplasmic E-cadherin-GFP intensity (mean ± SEM). Each dot represents one cell. A two-tailed one-way ANOVA test with Sidak correction was used. **c** Additional TP53^{R175H} mutation restores membrane E-cadherin levels. Representative confocal images show mosaically introduced cells with mKO2 alone (control) or with Ras^{G12V}, or both Ras^{G12V} and TP53^{R175H} (GFP^{+}, Ras^{+}, or Ras^{+}TP53^{RH+} cells) (magenta) in Tg(*krt4p:gal4*; UAS:zE-cadherin-GFP) larvae, which expressed Ecad-GFP ubiquitously in the whole skin. Scale bar: 50 µm. Right graphs show the ratio of membrane/cytoplasmic E-cadherin-GFP intensity (mean ± SEM). Each dot represents one cell. A two-tailed one-way ANOVA test with Sidak correction was used. **d** E-cadherin levels of cell mass is heterogeneous. Representative confocal images show mosaic Ras^{G12V} TP53^{R175H} cells (Ras^{+}TP53^{RH+} cell) (magenta) in Tg(*krt4p:gal4*; UAS:zE-cadherin-GFP) larvae. Yellow arrowheads and white arrowheads indicate E-cadherin-expressing Ras^{+}TP53^{RH+} cells and neighbouring cells, respectively. Yellow arrows and white arrows indicate Ras^{+}TP53^{RH+} cells and neighbouring cells lacking E-cadherin expression, respectively. Scale bar: 50 µm. Source data are provided as a Source Data file.

Supplementary Fig. 6

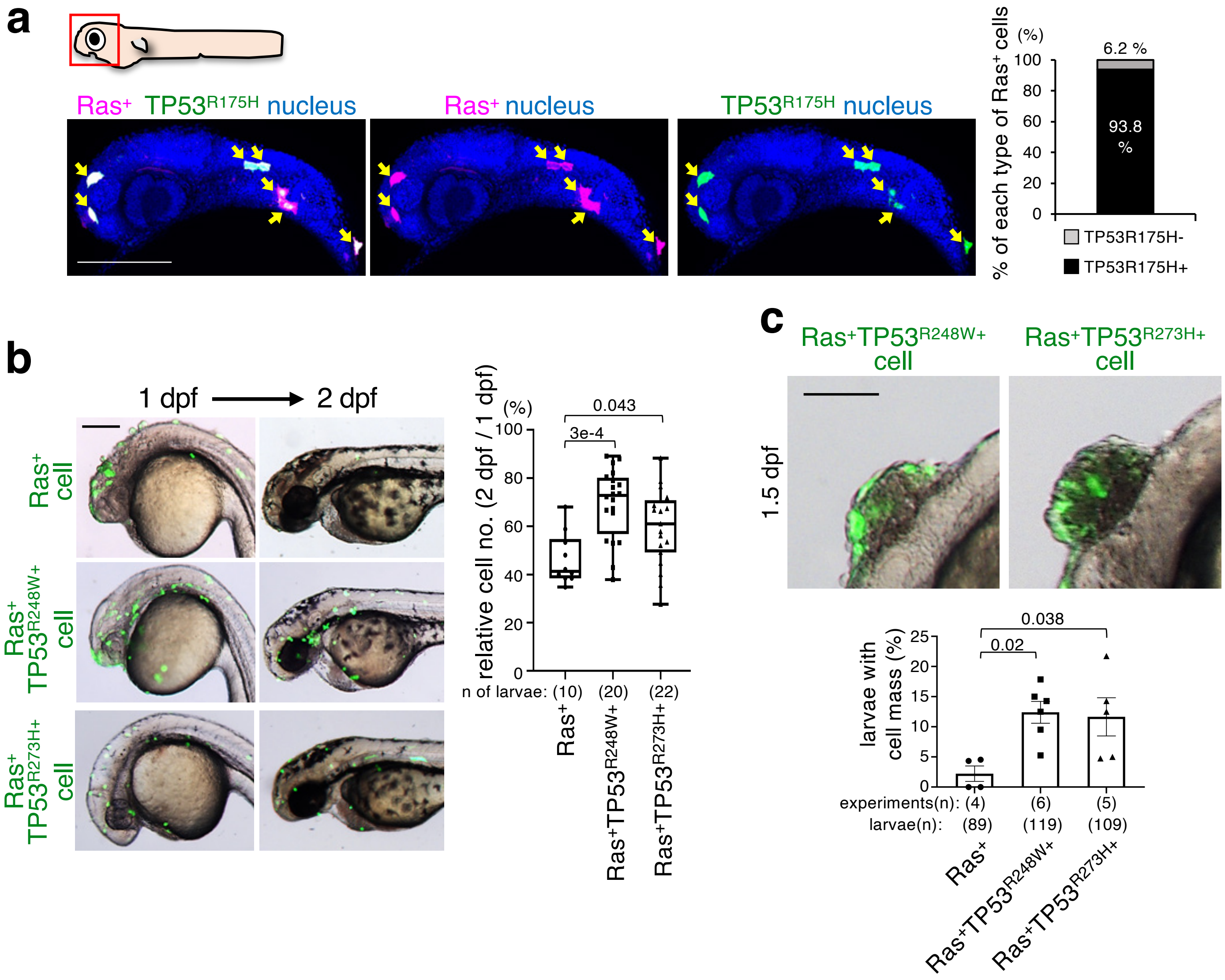

Supplementary Fig. 6. Co-introduction of TP53 gain-of function mutation blocks Ras^{G12V} cell elimination and stimulates cell mass formation.

a Co-introduction of both Ras^{G12V} and $TP53^{R175H}$ induces co-expression within single cells. Representative confocal image shows that $mCherry^{+}$ Ras^{G12V} (Ras^{+}) expression (magenta) and GFP^{+} $TP53^{R175H}$ ($TP53^{R175H+}$) expression (green) overlaps. Scale bar: 200 μm. 100% stacked bar graph shows percentage of total Ras^{G12V} cells. **b** Mosaically introduced-$Ras^{G12V}TP53^{R248W}$ cells and $Ras^{G12V}TP53^{R273H}$ cells remain in larval skin. Representative fluorescent and bright field (BF) images show 1 and 2 dpf larvae with mosaically introduced cells with GFP with Ras^{G12V}, both Ras^{G12V} and $TP53^{R248W}$, or both Ras^{G12V} and $TP53^{R273H}$ (Ras^{+}, $Ras^{+}TP53^{R248W+}$, or $Ras^{+}TP53^{R273H+}$ cells) (green) in the larvae. Scale bar: 200 μm. Box plots of relative GFP^{+} cell number of 2 dpf/1 dpf ratio show first and third quartile, median is represented by a line, whiskers indicate the minimum and maximum. Each dot represents one larva. A two-tailed one-way ANOVA test with Sidak correction was used. **c** Mosaically introduced $Ras^{G12V}TP53^{R248W}$ cells and $Ras^{G12V}TP53^{R273H}$ cells stimulate the cell mass formation. Representative images show cell mass in the head region of larvae with mosaically introduced $GFP^{+}Ras^{G12V}TP53^{R248W}$ or $GFP^{+}Ras^{G12V}TP53^{R273H}$ ($Ras^{+}TP53^{R248W+}$ or $Ras^{+}TP53^{R248W+}$ cells) (green) at 1.5 dpf. Scale bar: 200 μm. Bar graph shows the percentage of 1.5 dpf larvae with cell mass (mean ± SEM). Each dot represents an independent experimental result. A two-tailed one-way ANOVA test with Sidak correction was used. Source data are provided as a Source Data file.

Supplementary Fig. 7

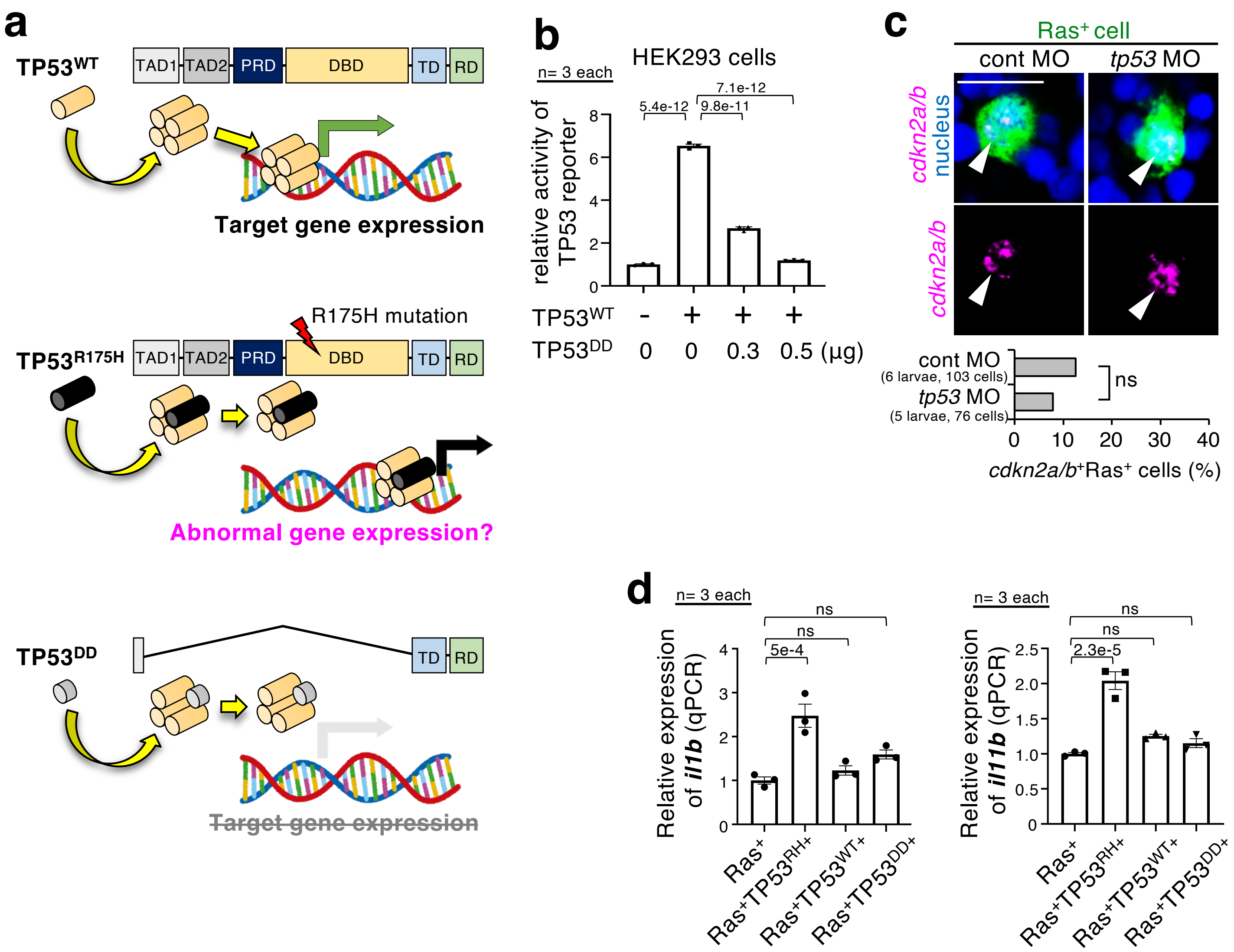

Supplementary Fig. 7. TP53 loss of function does not promote cellular senescence and SASP factor production.

a Schematic diagram shows structures and activities of TP53 wild-type ($TP53^{WT}$), a gain-of-function mutant ($TP53^{R175H}$), and dominant-negative mutant ($TP53^{DD}$). TAD: transactivation domain, PRO: proline-rich domain, DBD: DNA-binding domain, TD: tetramerization domain, RD: regulatory domain. $TP53^{WT}$ induces target gene expression. $TP53^{R175H}$ can induce abnormal gene expression. $TP53^{DD}$ can suppress target gene expression because of the loss of its TAD and DBD domains. **b** $TP53^{DD}$ functions as a dominant-negative mutant in culture cells. TP53 reporter plasmids and expression plasmids were transfected as indicated in HEK293 cells and luciferase activities were measured. $TP53^{DD}$ blocked $TP53^{WT}$-induced reporter activation in dose-dependent manner (mean ± SEM). Each dot represents an independent experimental result. A two-tailed one-way ANOVA test with Sidak correction was used. **c** Loss of TP53 does not promote oncogenic Ras-induced senescence. Representative confocal images show fluorescence *in situ* hybridization of *cdkn2a/b* mRNA (magenta) and nucleus (blue) in larvae with mosaically introduced cells expressing $GFP^{+}Ras^{G12V}$ cells (Ras^{+} cells) (green) injected with control MO or *tp53* MO. Arrowheads indicate *cdkn2a/b*$^{+}$ cells. Scale bar: 20 µm. Bottom graphs show percentages of Ras^{+} cells with *cdkn2a/b* expression. $p = 0.34$ (Fisher's exact test with Benjamini–Hochberg correction). **d** Additional $TP53^{R175H}$ mutation, but not $TP53^{WT}$ and $TP53^{DD}$, into Ras^{G12V} cells facilitate expression of SASP factors, *il1b* and *il11b*. qPCR analysis of expression of SASP factors (*il1b* and *il11b*) (mean ± SEM). Each dot represents an independent experimental result. A two-tailed one-way ANOVA test with Sidak correction was used. Source data are provided as a Source Data file.

Supplementary Fig. 8

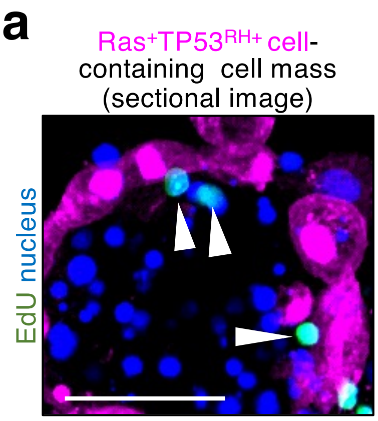

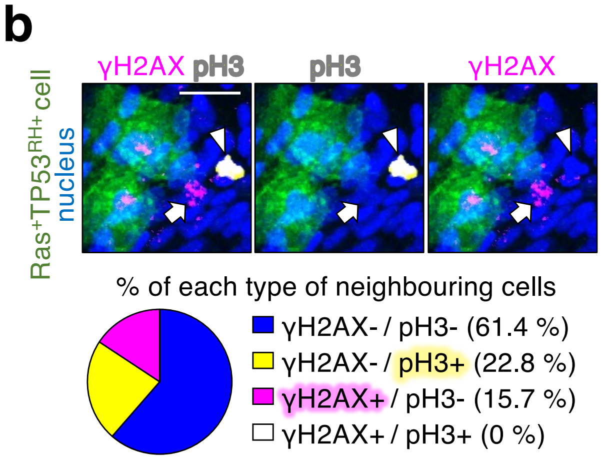

Supplementary Fig. 8. Proliferation and senescence in neighbours occur in a mutually exclusive manner.

a $Ras^{G12V}TP53^{R175H}$ cell-induced cell mass contains proliferative cells lacking $Ras^{+}TP53^{RH+}$ double mutation. Representative confocal images show that EdU^{+} proliferating cells (green) exist inside $mCherry^{+}Ras^{G12V+}TP53^{R175H}$ ($Ras^{+}TP53^{RH+}$) cell (magenta)-induced cell mass. Arrowheads indicate EdU^{+} cells lacking $Ras^{+}TP53^{RH+}$ double mutation. Scale bar: 50 µm. **b** Cell proliferation and senescence occur in a mutually exclusive manner. Representative confocal images show $\gamma H2AX^{+}$ senescent cells (magenta) and $pH3^{+}$ proliferating cells (yellow) exist in neighbouring area of $GFP^{+}Ras^{G12V}TP53^{R175H}$ ($Ras^{+}TP53^{RH+}$) cells (green). Arrows and arrowheads indicate $\gamma H2AX^{+}$ and $pH3^{+}$ cells, respectively. Scale bar: 20 µm. Pie chart indicates % of each type of cells. Note that there was no γH2AX and pH3 double-positive cells in larvae with mosaically introduced $Ras^{G12V}TP53^{R175H}$ cells. Source data are provided as a Source Data file.

Supplementary Fig. 9

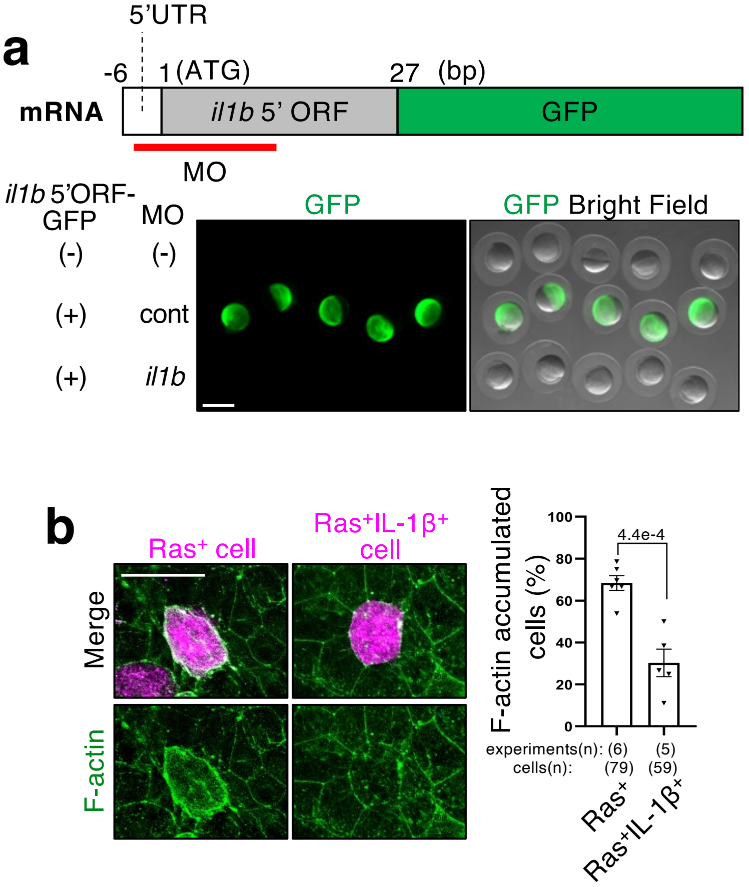

Supplementary Fig. 9. IL-1β affects behaviour of mosaically introduced Ras^{G12V} cells.

a *il1b* MO blocks translation of *il1b*. To examine the effect of *il1b* MO, mRNA including the 5′ untranslated region (UTR) and 5′ coding region (1–27 bp) of *il1b* fused in-frame with GFP (*il1b* 5′ open reading frame (ORF)-GFP) was injected into zebrafish embryos with control (cont) MO or *il1b* MO. The *il1b* MO annealing site is indicated by a red line. Representative images show that injection of *il1b* MO, but not cont MO, strongly diminished translation of *il1b* 5′ ORF-GFP mRNA. Scale bar: 500 µm. **b** Co-expression of exogenous Il1b in Ras^{G12V} cells reduced F-actin accumulation in the neighbouring cells. To generate Ras^{G12V} cells overexpressing Il1b, UAS-driven plasmid encoding zebrafish Il1b was co-introduced into Ras^{G12V} cells. Representative confocal images show F-actin (green) accumulation around mKO2^{+}Ras^{G12V} (Ras^{+}) cells or Ras^{+} cells overexpressing Il1b (Ras^{+}Il1b^{+}) (magenta). Scale bar: 20 µm. Right graphs show percentage of F-actin accumulated Ras^{G12V+} cells (mean ± SEM). Each dot represents an independent experimental result. Unpaired two-tailed t-test was used. Source data are provided as a Source Data file.

Supplementary Fig. 10

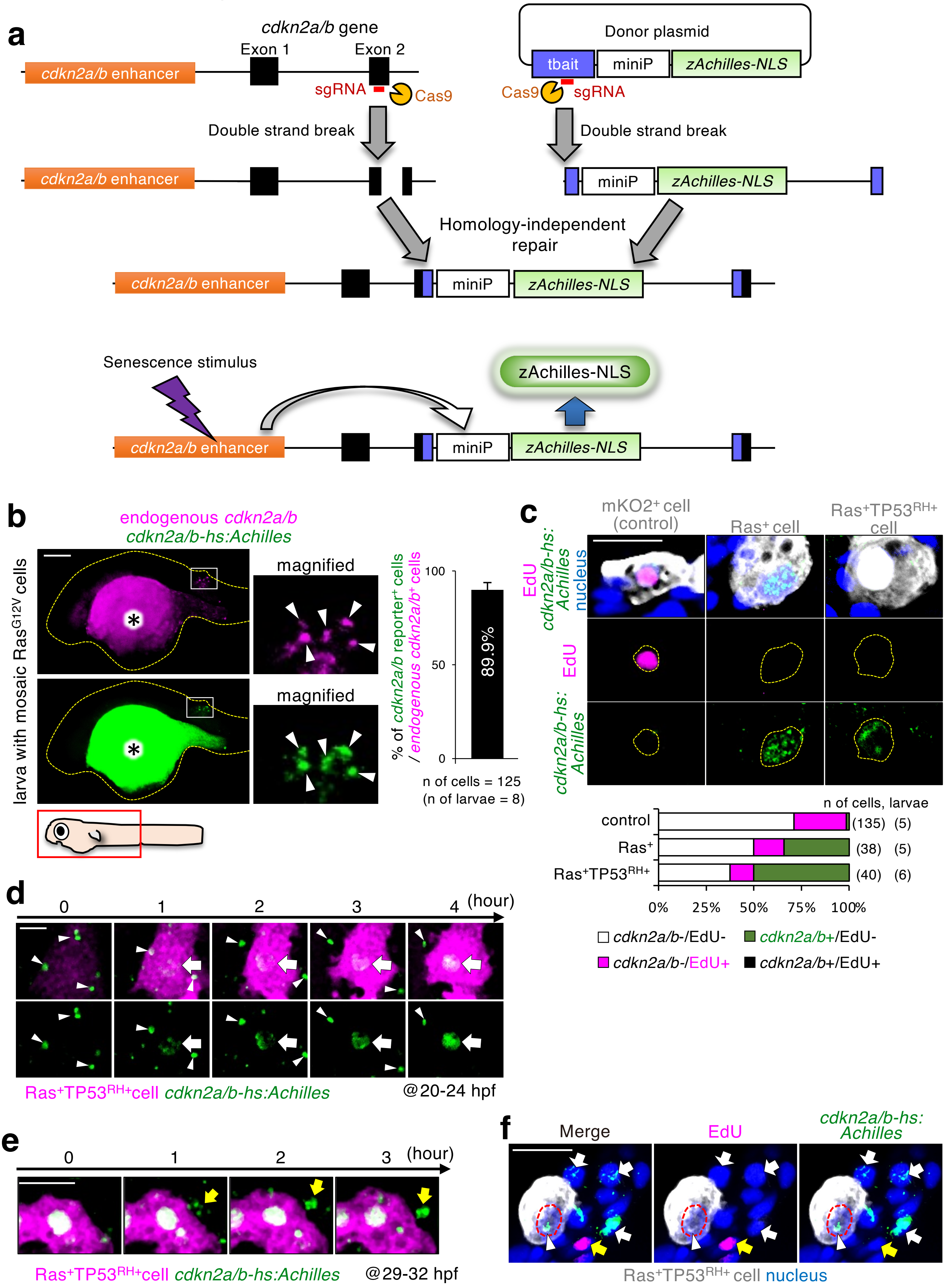

Supplementary Fig. 10. *cdkn2a/b* reporter visualizes senescent cells in zebrafish.

a Schematic diagram of Tg(*cdkn2a/b*-hs:Achilles) zebrafish generation. **b** *cdkn2a/b*-hs:Achilles reporter reflects the endogenous *cdkn2a/b* expression in zebrafish larvae. Representative images show fluorescent *in situ* hybridization of endogenous *cdkn2a/b*-expression (magenta). Introduction of mosaic Ras^{G12V} mutation induced Achilles expression (green) in endogenous *cdkn2a/b*-expressing cells in Tg(*cdkn2a/b*-hs:Achilles) larvae. Dot lines surround larvae. Boxed area magnified (white line). Arrowheads indicate *cdkn2a/b* reporter and endogenous *cdkn2a/b* double-positive cells. Asterisks indicate non-specific fluorescence in yolk sac. Scale bar: 200 μm. Right bar graph shows percentages of *cdkn2a/b* $reporter^{+}$ cells/endogenous *cdkn2a/b*$^{+}$ cells. **c** Cell-cycle in *cdkn2a/b* reporter-expressing cells is arrested. Representative images show *cdkn2a/b* $reporter^{+}$ senescent cells (green), EdU^{+} proliferating cells (magenta), and nucleus (blue) in Tg(*cdkn2a/b*-hs:Achilles) larvae with mosaically introduced $mKO2^{+}$ cells (control), Ras^{G12V} (Ras^{+}) cells, $Ras^{G12V}TP53^{R175H}$ ($Ras^{+}TP53^{RH+}$) cells (grey). Dot lines surround nucleus of $mKO2^{+}$ cell, Ras^{+} cell, and $Ras^{+}TP53^{RH+}$ cell. Scale bar: 20 μm. Bottom 100% stacked bar graph shows percentage of *cdkn2a/b* reporter- and/or EdU- positive (+) and negative (-) cells. Note that *cdkn2a/b* reporter and EdU double-positive cells were not detected. **d**, **e** Live imaging of $Ras^{G12V}TP53^{R175H}$ ($Ras^{+}TP53^{RH+}$) cell and neighbouring cells to senesce. Representative images show *cdkn2a/b* expression in $Ras^{+}TP53^{RH+}$ cell (**d**, arrows) and neighbouring cells (**e**, arrows). Arrowheads indicate non-specific fluorescence. Scale bar: 20 μm. See also Supplementary Movies 4 and 5. **f** Cell proliferation and senescence occur in a mutually exclusive manner in *cdkn2a/b* reporter larvae. Representative images show *cdkn2a/b* $reporter^{+}$ senescent cells (green), EdU^{+} proliferating cells (magenta), and nucleus (blue) in Tg(*cdkn2a/b*-hs:Achilles) larvae with mosaically introduced $mCherry^{+}Ras^{G12V}TP53^{R175H}$ ($Ras^{+}TP53^{RH+}$) cells (grey). White arrowhead and white arrows indicate *cdkn2a/b*$^{+}$ $Ras^{+}TP53^{RH+}$ cell and neighbouring cells, respectively. Yellow arrow indicates EdU^{+} proliferating cells. Scale bar: 20 μm. Source data are provided as a Source Data file.

Supplementary Fig. 11

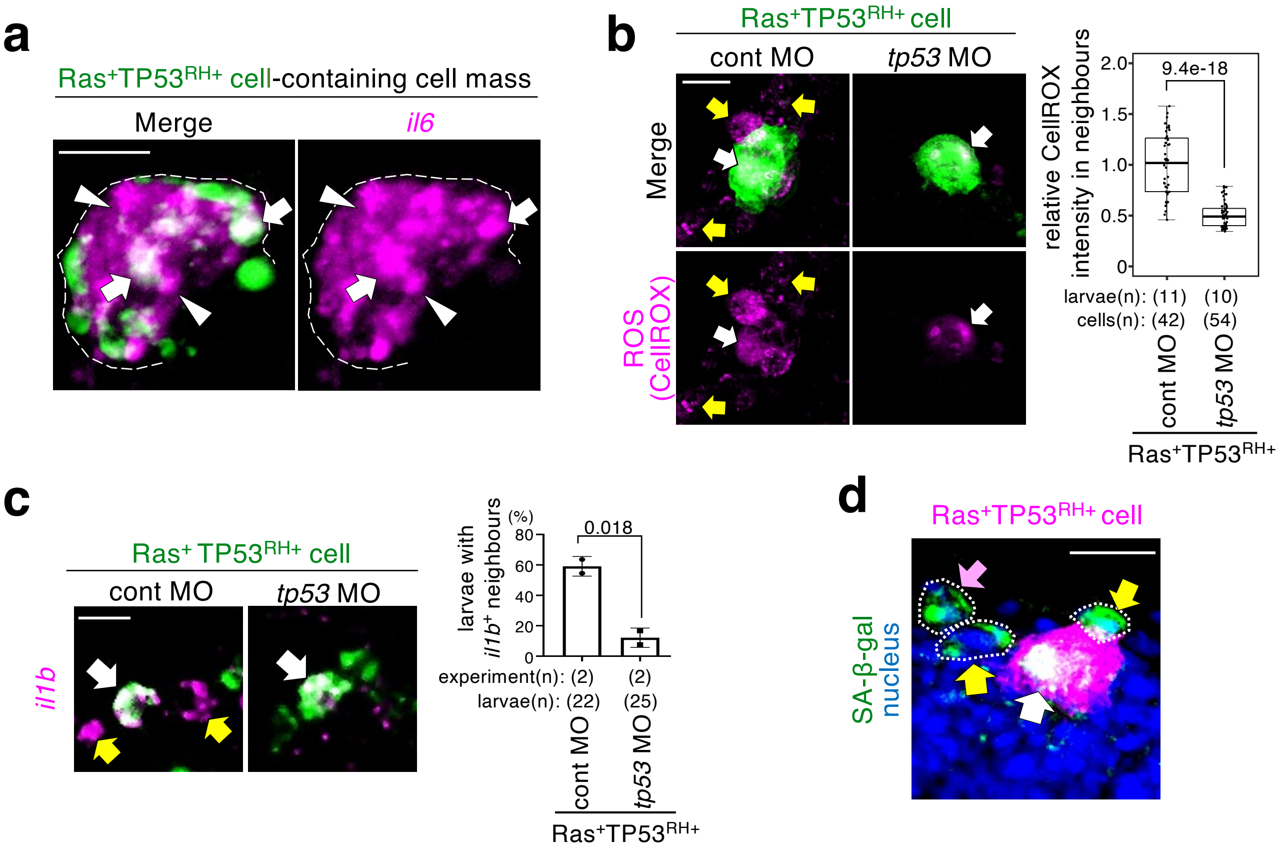

Supplementary Fig. 11. SASP factor production in neighbouring cells depends on *tp53* activity.

a SASP factor *il6* is expressed in neighbouring cells and $Ras^{+}TP53^{RH+}$ cells within the cell mass. Representative images show fluorescent *in situ* hybridization of *il6* (magenta) in a cell mass formed in the head region of larvae with mosaically introduced cells expressing $GFP^{+}Ras^{+}TP53^{RH+}$ ($Ras^{+}TP53^{RH+}$) (green). Dotted lines surround cell mass. Arrows indicate *il6*-expressing $Ras^{+}TP53^{RH+}$ cells. Arrowheads indicate *il6*-expressing neighbours. Scale bar: 50 µm. **b** Production of ROS in neighbours of $Ras^{+}TP53^{RH+}$ cells is dependent on *tp53* activity. Representative confocal images show ROS-probe (CellROX DeepRed (magenta))-stained larvae with mosaically introduced cells expressing $GFP^{+}Ras^{+}TP53^{RH+}$ ($Ras^{+}TP53^{RH+}$) (green) with control MO or *tp53* MO injection. White arrows indicate ROS in $Ras^{+}TP53^{RH+}$ cells. Yellow arrows indicate ROS in the neighbouring area. Scale bar: 20 µm. Box plots of relative CellROX intensities (normalized by that of control MO) in neighbouring area (20 µm radius around $Ras^{+}TP53^{RH+}$ cells) show first and third quartile, median is represented by a line, whiskers indicate the minimum and maximum. Each dot represents one area. Unpaired two-tailed t-test was used. **c** Expression of *il1b* in neighbours of $Ras^{+}TP53^{RH+}$ cells is dependent on *tp53* activity. Representative images show fluorescent *in situ* hybridization of *il1b* (magenta) in larvae with mosaic $GFP^{+}Ras^{+}TP53^{RH+}$ cells (green) with control MO or *tp53* MO. White arrows indicate *il1b*-expressing $Ras^{+}TP53^{RH+}$ cells. Yellow arrows indicate *il1b*-expressing neighbours. Scale bar: 20 µm. Bar plots show percentage of larvae with $il1b^{+}$ neighbours (mean ± SEM). Each dot represents an independent experimental result. Unpaired two-tailed t-test was used. **d** SA-β-gal activity is detected in not only the area immediately neighbouring the double-mutant cells but also 2 cells away from these cells. Representative confocal images show mosaically introduced $mCherry^{+}Ras^{+}TP53^{RH+}$ cell (magenta), SA-β-gal (green), and nucleus (blue). Dotted line indicates SA-β-gal^{+} neighbours. White arrow, yellow arrow, and pink arrow indicate SA-β-$gal^{+}Ras^{+}TP53^{RH+}$ cells, adjacent neighbours, second adjacent neighbours, respectively. Scale bar: 20 µm. Source data are provided as a Source Data file.

Supplementary Fig. 12

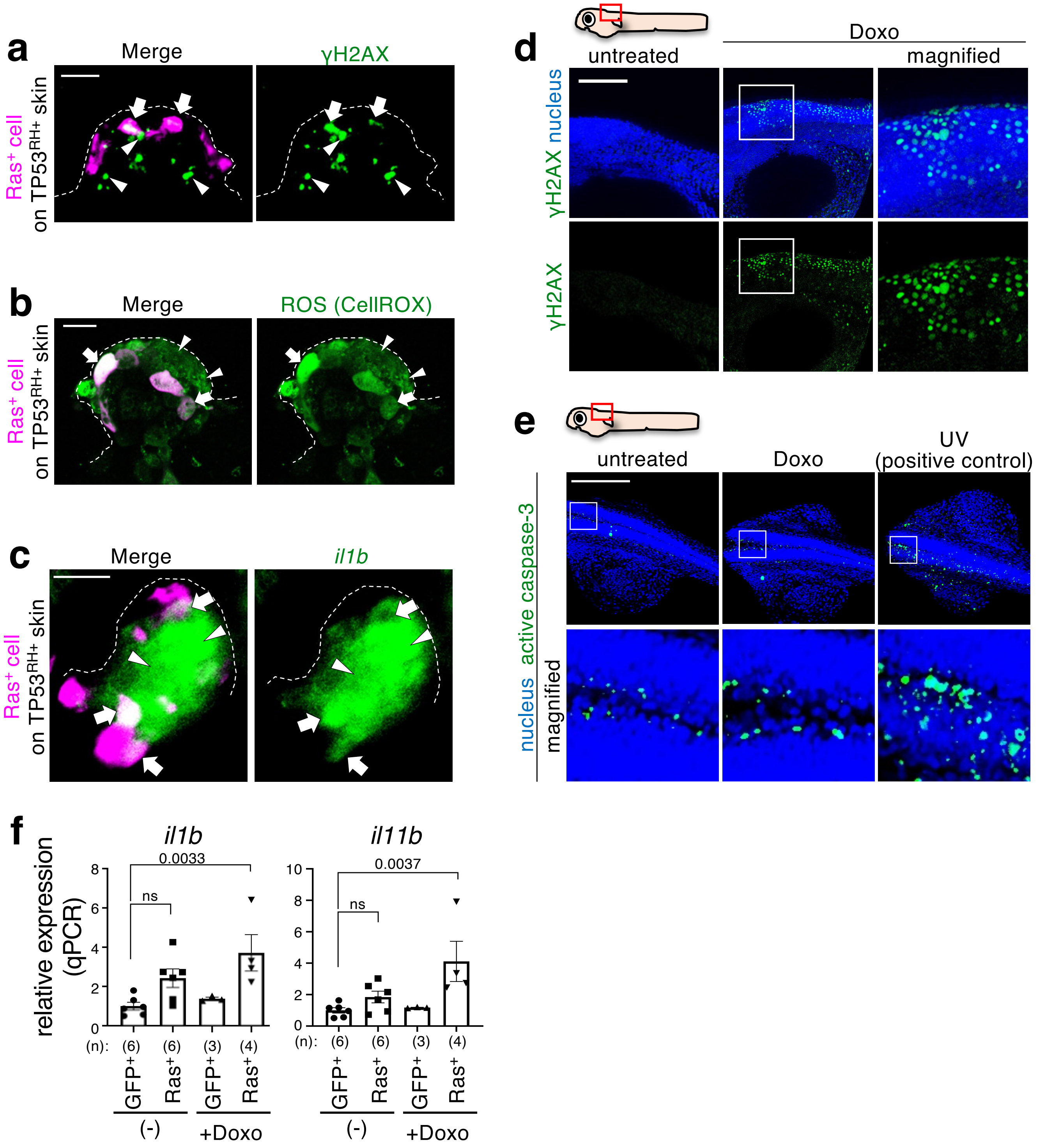

Supplementary Fig. 12. Mosaic Ras^{G12V} cells form a cell mass by producing SASP factors in damaged skin.

a–c Ras^{G12V} cells stimulates senescence and SASP factor production in neighbours on TP53^{R175H}-expressing skin. Representative images show immunostaining of γH2AX (**a**), CellROX-detected ROS (**b**), or *in situ* hybridization of *il1b* mRNA (**c**) (green) in a cell mass formed in the trunk (**a**, **b**) or tail (**c**) region of Tg(*krt4p:gal4*; UAS:GAP43EGFP-T2A-TP53^{R175H}) larvae mosaically introduced Ras^{G12V} mutation and mCherry (Ras^{+}) (magenta). Dotted lines surround cell mass. Scale bar: 50 µm. **d,e** Doxorubicin treatment induces DNA damage in part of cells but does not induce apoptosis. In **d**, representative confocal images show γH2AX (green) and nucleus (blue) in larvae untreated or doxorubicin (Doxo). Boxed area magnified (white line) to show γH2AX induced in part of the skin. Scale bar: 200 µm. In **e**, representative confocal images show active caspase-3 (green) and nucleus (blue) in larvae untreated, treated with Doxo or UV. Boxed area magnified (white line). As a positive control for active caspase-3 detection larvae were exposed to UV (60 mJ/cm^2) and incubated for 5 h at 28.5°C. UV treatment increased active caspase-3 positive apoptotic cells, whereas Doxo did not. Scale bar: 200 µm. **f** Doxorubicin treatment upregulates expression of SASP factors, *il1b* and *il11b*, in larvae with mosaically introduced Ras^{G12V} cells. Graphs show qPCR analysis for *il1b* and *il11b* (mean ± SEM). Each dot represents an independent experimental result. A two-tailed one-way ANOVA test with Sidak correction was used. Source data are provided as a Source Data file.

Supplementary Fig. 13

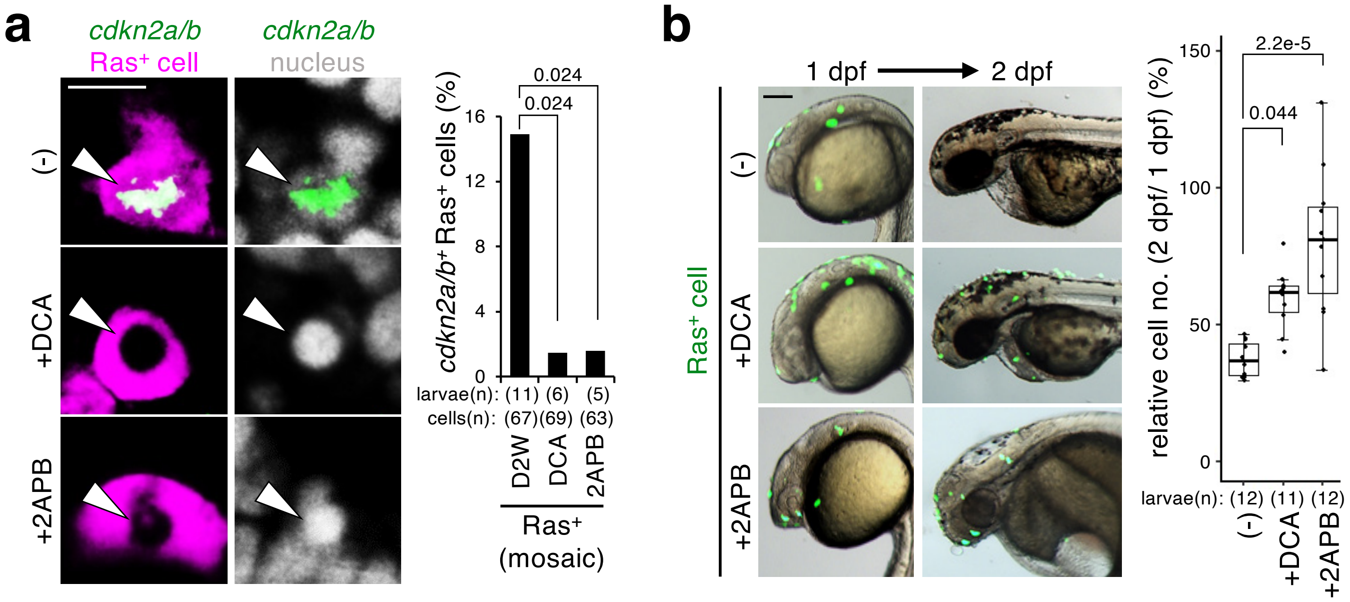

Supplementary Fig. 13. EDAC mediators are involved in non-cell autonomous senescence of mosaic Ras^{G12V} cells.

a,b Inhibition of EDAC mediators prevent cellular senescence (**a**) and elimination (**b**) of Ras^{G12V} cell elimination. In **a**, representative images show fluorescent *in situ* hybridization for *cdkn2a/b* (green) in larvae with mosaically introduced-$GFP^{+}Ras^{G12V}$ (Ras^{+}) cells (magenta) treated with or without DCA (inhibitor of PDK family) or 2APB (inhibitor of mechanosensitive calcium channel and IP3 receptor). Scale bar: 20 µm. Bar graph show percentage of *cdkn2a/b*$^{+}$$Ras^{+}$ cells. Fisher's exact test with Benjamini–Hochberg correction was used. In **b**, representative fluorescent and bright field (BF) images show 1 and 2 dpf larvae with mosaically introduced cells with $GFP^{+}Ras^{G12V+}$ (Ras^{+} cells) (green) in larvae treated with or without DCA or 2APB. Scale bar: 200 µm. Box plots of relative GFP^{+} cell number of 2 dpf/1 dpf ratio show first and third quartile, median is represented by a line, whiskers indicate the minimum and maximum, and outliers are shown as dots outside of the box. Each dot represents one larva. A two-tailed one-way ANOVA test with Sidak correction was used. Source data are provided as a Source Data file.

Supplementary Table 1

RNA Probe primers

mKO2 (Fw)	5'-ACT ACA TGG ACG GCT CCG TC-3'
mKO2 (RV)	5'-AGC CTG TGG CCG ATG TAG T-3'
zebrafish-codon-optimized *Achilles* (Fw)	5'-ATG GTA AGT AAA GGT GAA G-3'
zebrafish-codon-optimized *Achilles* (Rv)	5'-TTT GTA CAG CTC GTC CA-3'
il1b (Fw)	5'-GAG CTA CAG ATG CGA CAT GC-3'
il1b (Rv)	5'-AGT ACG AGA TGT GGA GCG GA-3'
il6 (Fw)	5'-TCG CTG ACC CGG TCC CCG-3'
il6 (Rv)	5'-CAA CTA CAT GAG CAG GAG G-3'
cxcl8a (Fw)	5'-CAT GTT GAC AGA ATT CAC CAG CTG-3'
cxcl8a (Rv)	5'-GCA GGA TTT ATG TGA ACA ACA CTC AT-3'
il11b (Fw)	5'-TGT GCT AAC AGT GTC GCC TGA-3'
il11b (Rv)	5'-CTG GCA CCA TAA AGA TTG GCT GA-3'
cdkn2a/b (Fw)	5'-CAG CGT TGA ACT GAT TGT TTT CGC A-3'
cdkn2a/b (Rv)	5'-GAC TGC ATC ACT GCA TAG CCT AGG-3'

RT-qPCR primers

actb1 (Fw)	5'-TGG ACT TTG AGC AGG AGA TGG GAA-3'
actb1 (Rv)	5'-AAG GTG GTC TCA TGG ATA CCG CAA-3'
il1b (Fw)	5'-ATC AAA CCC CAA TCC ACA GAG T-3'
il1b (Rv)	5'-GGC ACT GAA GAC ACC ACG TT-3'
il11b (Fw)	5'-CAT CTT ATC CAA GCT ATC ATC CAG-3'
il11b (Rv)	5'-GAT CTC GGG TGC TGT CTG TC-3'

MO

		references
Standard control (5 ng)	5'-CCT CTT ACC TCA GTT ACA ATT TAT A-3'	
il1b (5 ng)	5'-CAT ATT GCC CGC ATG CCA TCA TTT C-3'	This paper
spi1b (5 ng)	5'-GAT ATA CTG ATA CTC CAT TGG TGG T-3'	#33 (Rhodes et al., 2005)
tp53 (2.5-5 ng)	5'-GCG CCA TTG CTT TGC AAG AAT TG-3'	#93 (Robu et al., 2007)
cdkn2a/b (2.5 ng)	5'-TAA AGC GCG TCT AAA CCT ACC TGT A-3'	#94 (Mochida et al., 2012)

sgRNA

cdkn2a/b (Exon 2)	5'-GAG GTG TGC TCC CTG TGT CG**G GG**-3'
Tbait	5'-GGC TGC TGT CAG GGA GCT CA**T GG**-3'

antibodies

**Primary antibodies**	calatog number, maker, ditution
Rabbit anti-γH2AX	#2577, Cell Signaling Technology, Mountain View, CA, 1/500
Mouse anti-γH2AX	#630856, MERCK Millipore, Burlington, MA, 1/100
Rabbit anti-trimethyl-Histone H3 (Lys9)	#07-442, MERCK Millipore, Burlington, MA, 1/100
Rabbit anti-pH3	#06-570, MERCK Millipore, Burlington, MA, 1/200
Rabbit anti-active caspase-3	#559565, BD Bioscience, USA, 1/300
Mouse anti-mKO2	#M168-3M, MBL, Nagoya, Japan, 1/800
Rabbit anti-mKO2	#PM051M, MBL, Nagoya, Japan, 1/800
Rabbit anti-GFP	#A-11122, Thermo Fisher, Waltham, MA, 1/500
Chicken anti-GFP	#ab13970, abcam, Cambridge, UK, 1/800
Rabbit anti-DsRed	#632496, Takara Bio, Kusatsu, Japan, 1/1000
**Secondary antibodies**	
AlexaFluor488-conjugated anti-mouse IgG	#A-11029, Invitrogen, Waltham, MA, 1/300
AlexaFluor488-conjugated anti-rabbit IgG	#A-11034, Invitrogen, Waltham, MA, 1/300
AlexaFluor488-conjugated anti-chicken IgY	#A-11039, Invitrogen, Waltham, MA, 1/300
AlexaFluor594-conjugated anti-mouse IgG	#A-11032, Invitrogen, Waltham, MA, 1/300
AlexaFluor594-conjugated anti-rabbit IgG	#A-11037, Invitrogen, Waltham, MA, 1/300
AlexaFluor647-conjugated anti-mouse IgG	#A32728, Invitrogen, Waltham, MA, 1/300
AlexaFluor647-conjugated anti-rabbit IgG	#4414, Cell Signaling Technology, Mountain View, CA, 1/300
