# Supplementary material for: Zebrafish imaging reveals TP53 mutation switching oncogene-induced senescence from suppressor to driver in primary tumorigenesis
Source: Nat Commun. 2022 Mar 18;13:1417. doi: 10.1038/s41467-022-29061-6 (PMC8933407; doi:10.1038/s41467-022-29061-6)
Supplement: Supplementary file 3 — Description of Additional Supplementary Files [file 41467_2022_29061_MOESM3_ESM.pdf]

Description of Additional Supplementary Files

File Name: Supplementary Movie 1 & 2
Description: **Mosaic Ras^{G12V} cell apically protrudes (1) and leaves (2) from the larval skin.**
Time-lapse imaging of the head region of 1 dpf Tg(*krt4p:gal4;* UAS:EGFP) larva with mosaically introduced $mKO2^+$ Ras^+ cells (red).

File Name: Supplementary Movie 3
Description: **Mosaic Ras^{G12V}-$TP53^{R175H}$ double-mutant cells forms heterogenous tumour-like cell mass.**
Time-lapse imaging of the head region of 1 dpf Tg(*krt4p:gal4;* UAS:EGFP) larva with mosaically introduced $mKO2^+Ras^+TP53^+$ cells (red).

File Name: Supplementary Movie 4
Description: **Live imaging of Ras^{G12V}-$TP53^{R175H}$ double-mutant cells to senesce.**
Time-lapse imaging of *cdkn2a/b* reporter expression (green) in 20−24 hpf Tg(*cdkn2a/b*-hs:Achilles) larvae with mosaically introduced $mCherry^+Ras^{G12V}TP53^{R175H}$ cells (magenta). *cdkn2a/b* reporter was gradually upregulated in the nucleus of double-mutant cell.

File Name: Supplementary Movie 5
Description: **Live imaging of Ras^{G12V}-$TP53^{R175H}$ double-mutant cell neighbours to senesce.**
Time-lapse imaging of *cdkn2a/b* reporter expression (green) in 29−32 hpf Tg(*cdkn2a/b*-hs:Achilles) larvae with mosaically introduced $mCherry^+Ras^{G12V}TP53^{R175H}$ cells (magenta). *cdkn2a/b* reporter was gradually upregulated in the nucleus of double-mutant cell neighbour.
