# Supplementary material for: Zebrafish imaging reveals TP53 mutation switching oncogene-induced senescence from suppressor to driver in primary tumorigenesis
Source: Nat Commun. 2022 Mar 18;13:1417. doi: 10.1038/s41467-022-29061-6 (PMC8933407; doi:10.1038/s41467-022-29061-6)
Supplement: Supplementary file 9 — Reporting Summary [file 41467_2022_29061_MOESM9_ESM.pdf]

nature research

Corresponding author(s): Tohru Ishitani

Last updated by author(s): Feb 4, 2022

Reporting Summary

Nature Research wishes to improve the reproducibility of the work that we publish. This form provides structure for consistency and transparency in reporting. For further information on Nature Research policies, see our Editorial Policies and the Editorial Policy Checklist.

Statistics

For all statistical analyses, confirm that the following items are present in the figure legend, table legend, main text, or Methods section.

n/a	Confirmed	
☐	☒	The exact sample size (*n*) for each experimental group/condition, given as a discrete number and unit of measurement
☐	☒	A statement on whether measurements were taken from distinct samples or whether the same sample was measured repeatedly
☐	☒	The statistical test(s) used AND whether they are one- or two-sided<br>*Only common tests should be described solely by name; describe more complex techniques in the Methods section.*
☒	☐	A description of all covariates tested
☒	☐	A description of any assumptions or corrections, such as tests of normality and adjustment for multiple comparisons
☐	☒	A full description of the statistical parameters including central tendency (e.g. means) or other basic estimates (e.g. regression coefficient) AND variation (e.g. standard deviation) or associated estimates of uncertainty (e.g. confidence intervals)
☐	☒	For null hypothesis testing, the test statistic (e.g. *F*, *t*, *r*) with confidence intervals, effect sizes, degrees of freedom and *P* value noted<br>*Give P values as exact values whenever suitable.*
☒	☐	For Bayesian analysis, information on the choice of priors and Markov chain Monte Carlo settings
☒	☐	For hierarchical and complex designs, identification of the appropriate level for tests and full reporting of outcomes
☒	☐	Estimates of effect sizes (e.g. Cohen's *d*, Pearson's *r*), indicating how they were calculated

Our web collection on statistics for biologists contains articles on many of the points above.

Software and code

Policy information about availability of computer code

Data collection	Stereo microscope: MZ16FA (Leica)<br>Confocal laser-scanning microscope: LSM700 (Zeiss), FV3000 (Olympus)<br>qPCR: Mx3000P QPCR system (Agilent)
Data analysis	Imaging data analysis: Fiji/ImageJ, GIMP 2.10.18, Imaris (Bitplane)<br>Statistical analysis and graphs: Excel 2019 (Microsoft), R(The R Project for Statistical Computing), Prism8 (GraphPad)

For manuscripts utilizing custom algorithms or software that are central to the research but not yet described in published literature, software must be made available to editors and reviewers. We strongly encourage code deposition in a community repository (e.g. GitHub). See the Nature Research guidelines for submitting code & software for further information.

Data

Policy information about availability of data

All manuscripts must include a data availability statement. This statement should provide the following information, where applicable:

- Accession codes, unique identifiers, or web links for publicly available datasets
- A list of figures that have associated raw data
- A description of any restrictions on data availability

Raw data associated the figures are included in the Source Data file. The data that support the findings of this study are available from the corresponding author upon reasonable request.

Field-specific reporting

Please select the one below that is the best fit for your research. If you are not sure, read the appropriate sections before making your selection.

☒ Life sciences ☐ Behavioural & social sciences ☐ Ecological, evolutionary & environmental sciences

For a reference copy of the document with all sections, see nature.com/documents/nr-reporting-summary-flat.pdf

Life sciences study design

All studies must disclose on these points even when the disclosure is negative.

Sample size	No statistical methods were used to predetermined sample size. Sample size was chosen based on previous study used the similar methods (Akieda et al. "Cell competition corrects noisy Wnt morphogen gradients to achieve robust patterning in the zebrafish embryo." Nature communications vol. 10,1 4710. 17 Oct. 2019, doi:10.1038/s41467-019-12609-4).
Data exclusions	We excluded from our analyses unviable embryos. Miss injected embryos were also excluded from analysis.
Replication	At least two independent experiments were taken to verify the reproducibility of the experimental findings. All experiments were reliably reproduced.
Randomization	Embryos from zebrafish crosses were randomly chosen for the injection. Injected embryos were randomly allocated to experimental groups after sorting for expression of fluorescent proteins (e.g. GFP, mKO2).
Blinding	The investigators were not blinded to the groups and treatments during experiments,since embryos from zebrafish crosses were genetically uniform and indistinguishable.

Reporting for specific materials, systems and methods

We require information from authors about some types of materials, experimental systems and methods used in many studies. Here, indicate whether each material, system or method listed is relevant to your study. If you are not sure if a list item applies to your research, read the appropriate section before selecting a response.

Materials & experimental systems

n/a	Involved in the study	
☐	☒	Antibodies
☐	☒	Eukaryotic cell lines
☒	☐	Palaeontology and archaeology
☐	☒	Animals and other organisms
☒	☐	Human research participants
☒	☐	Clinical data
☒	☐	Dual use research of concern

Methods

n/a	Involved in the study	
☒	☐	ChIP-seq
☒	☐	Flow cytometry
☒	☐	MRI-based neuroimaging

Antibodies

Antibodies used	Primary antibodies were as follows: rabbit anti-γH2AX (#2577, Cell Signaling Technology, Mountain View, CA); mouse anti-γH2AX (#630856, MERCK Millipore, Burlington, MA); rabbit anti-pH3 (#06-570, MERCK Millipore, Burlington, MA); rabbit anti-trimethyl-Histone H3 (Lys9) (#07-442, MERCK Millipore, Burlington, MA); rabbit anti-active caspase-3 (#559565, BD Bioscience, USA); mouse anti-mKO2 (#M168-3M, MBL, Nagoya, Japan); rabbit anti-mKO2 (#PM051M, MBL, Nagoya, Japan); rabbit anti-GFP (#A-11122, Thermo Fisher, Waltham, MA); chicken anti-GFP (#ab13970, abcam, Cambridge, UK); rabbit anti-DsRed (#632496, Takara Bio, Kusatsu, Japan).<br>Secondary antibodies were as follows: AlexaFluor488-conjugated anti-mouse IgG (#A-11029, Invitrogen, Waltham, MA), anti-rabbit IgG (#A-11034, Invitrogen) and anti-chicken IgY (#A-11039, Invitrogen); AlexaFluor594-conjugated anti-mouse IgG (#A-11032, Invitrogen) and anti-rabbit IgG (#A-11037, Invitrogen); AlexaFluor647-conjugated anti-mouse IgG (#A32728, Invitrogen) and anti-rabbit IgG (#4414, Cell Signaling Technology, Mountain View, CA).
Validation	Only commercially available antibodies were used. They were all validated by the producers.<br>The validation statements on the manufacturer's website are as follows.<br>• rabbit anti-γH2AX (#2577); https://en.cellsignal.jp/products/primary-antibodies/phospho-histone-h2a-x-ser139-antibody/2577<br>• mouse anti-γH2AX (#5-636); https://www.merckmillipore.com/JP/ja/product/Anti-phospho-Histone-H2A.X-Ser139-Antibody-clone-JBW301,MM_NF-05-636#<br>• rabbit anti-pH3 (#06-570); https://www.merckmillipore.com/JP/ja/product/Anti-phospho-Histone-H3-Ser10-Antibody-Mitosis-Marker,MM_NF-06-570#<br>• rabbit anti-trimethyl-Histone H3 (Lys9) (#07-442); https://www.merckmillipore.com/JP/ja/product/Anti-trimethyl-Histone-H3-Lys9-Antibody,MM_NF-07-442#overview

• rabbit anti-active caspase-3 (#559565); https://www.bdbiosciences.com/en-us/products/reagents/flow-cytometry-reagents/research-reagents/single-color-antibodies-ruo/purified-rabbit-anti-active-caspase-3.559565
• mouse anti-mKO2 (#M168-3M); https://ruo.mbl.co.jp/bio/dtl/A/?pcd=M168-3M
• rabbit anti-mKO2 (#PM051M); https://ruo.mbl.co.jp/bio/dtl/A/?pcd=PM051M
• rabbit anti-GFP (#A-11122); https://www.thermofisher.com/antibody/product/GFP-Antibody-Polyclonal/A-11122
• chicken anti-GFP (#ab13970); https://www.abcam.co.jp/gfp-antibody-ab13970.html
• rabbit anti-DsRed (#632496); https://www.takarabio.com/products/antibodies-and-elisa/fluorescent-protein-antibodies/red-fluorescent-protein-antibodies

Eukaryotic cell lines

Policy information about cell lines

Cell line source(s)

HEK293 cell line was gifted form Dr. Kunihiro Matsumoto. Original commercial source was #CRL-1573™, ATCC.

Authentication

To authenticate HEK293 cells, we performed periodic morphology check via microscope. To prevent deterioration of cells, we used the cells at low passage numbers.

Mycoplasma contamination

Cell line tested negative for mycoplasma contamination.

Commonly misidentified lines
(See ICLAC register)

No commonly misidentified cell lines were used.

Animals and other organisms

Policy information about studies involving animals; ARRIVE guidelines recommended for reporting animal research

Laboratory animals

Adults zebrafish (Danio rerio) were used to obtain fertilized eggs. Zebrafish larvae used in this study were at 1 to 2 day post fertilization, respectively. Sex is not yet determined in zebrafish larvae at this stage.

• Zebrafish wild-type strain (AB),
• Tg(krt4p:gal4; UAS:EGFP),
• Tg(krt4p:gal4; UAS:mKO2),
• Tg(UAS:GAP43mKO2-T2A-H-RasG12V),
• Tg(UAS:GAP43EGFP-T2A-TP53R175H),
• Tg(cdkn2a/b-hs:Achilles),
• Tg(UAS:zE-cadherin-GFP),
• Tg(il1b:EGFP)

Wild animals

We did not use wild animals.

Field-collected samples

We did not use field-collected samples.

Ethics oversight

All experimental animal care was performed in accordance with institutional and national guidelines and regulations. The study protocol was approved by the Institutional Animal Care and Use Committee of the respective universities (Osaka University, RIMD Permit# R02-04; Gunma University Permit# 17-051).

Note that full information on the approval of the study protocol must also be provided in the manuscript.
